# Nasopharyngeal microbiota in hospitalized children with *Bordetella pertussis* and Rhinovirus infection

**DOI:** 10.1038/s41598-021-02322-y

**Published:** 2021-11-24

**Authors:** A. E. Tozzi, F. Del Chierico, E. Pandolfi, S. Reddel, F. Gesualdo, S. Gardini, V. Guarrasi, L. Russo, I. Croci, I. Campagna, G. Linardos, C. Concato, A. Villani, L. Putignani

**Affiliations:** 1 Multifactorial Disease and Complex Phenotype Research Area, Bambino Gesù Children’s Hospital, IRCCS, Piazza S. Onofrio, 4, 00165 Rome, Italy; 2 Multimodal Laboratory Medicine Research Area, Unit of Human Microbiome, Bambino Gesù Children’s Hospital, IRCCS, Viale di San Paolo, 15, 00146 Rome, Italy; 3 GenomeUp SRL, Viale Pasteur, 6, 00144 Rome, Italy; 4 Department of Diagnostic and Laboratory Medicine, Unit of Microbiology and Diagnostic Immunology, Unit of Virology, Bambino Gesù Children’s Hospital, IRCCS, Piazza S. Onofrio, 4, 00165 Rome, Italy; 5 Department of Pediatrics, Bambino Gesù Children’s Hospital, IRCCS, Piazza S. Onofrio, 4, 00165 Rome, Italy; 6 Department of Diagnostic and Laboratory Medicine, Unit of Microbiology and Diagnostic Immunology, Unit of Microbiomics and Multimodal Laboratory Medicine Research Area, Unit of Human Microbiome, Bambino Gesù Children’s Hospital, IRCCS, Piazza S. Onofrio, 4, 00165 Rome, Italy

**Keywords:** Microbiology, Diseases, Medical research

## Abstract

Despite great advances in describing *Bordetella pertussis* infection, the role of the host microbiota in pertussis pathogenesis remains unexplored. Indeed, the microbiota plays important role in defending against bacterial and viral respiratory infections. We investigated the nasopharyngeal microbiota in infants infected by *B. pertussis* (Bp), Rhinovirus (Rv) and simultaneously by both infectious agents (Bp + Rv). We demonstrated a specific nasopharyngeal microbiome profiles for Bp group, compared to Rv and Bp + Rv groups, and a reduction of microbial richness during coinfection compared to the single infections. The comparison amongst the three groups showed the increase of Alcaligenaceae and *Achromobacter* in Bp and Moraxellaceae and *Moraxella* in Rv group. Furthermore, correlation analysis between patients’ features and nasopharyngeal microbiota profile highlighted a link between delivery and feeding modality, antibiotic administration and *B. pertussis* infection. A model classification demonstrated a microbiota fingerprinting specific of Bp and Rv infections. In conclusion, external factors since the first moments of life contribute to the alteration of nasopharyngeal microbiota, indeed increasing the susceptibility of the host to the pathogens' infections. When the infection is triggered, the presence of infectious agents modifies the microbiota favoring the overgrowth of commensal bacteria that turn in pathobionts, hence contributing to the disease severity.

## Introduction

The nasopharyngeal tract is inhabited by specific ecosystems that may change during infections^1–4^ and that play a central role in the host's susceptibility to pathogens^5^. However, as the microbiota in this district is strongly influenced by the environment, its microbial ecology and the interactions with pathogens are of particular interest in full comprehension of diseases.

*Bordetella pertussis* is the main causative agent of pertussis, a highly contagious respiratory infectious disease that continues to occur worldwide, in both highly vaccinated and unvaccinated populations^6^. Pertussis is still a major cause of morbidity and mortality in infants and children < 5 years old^7^. It is estimated that ~ 53,500 (0.9%) of the 5.941 million deaths in 2015 were due to pertussis in this age group^8^.

A significant proportion of pertussis infections presents with a concomitant viral infection, mostly represented by rhinoviruses^9,10^. While the clinical significance and pathogenesis of coinfections are not definitely clarified yet, either *B. pertussis* and rhinovirus infections in infancy have been associated with wheezing and asthma in older ages^11,12^. The pathogenesis of asthma episodes may involve the ecology of the respiratory tract as suggested by some authors^3,13,14^.

Modulation of the nasopharyngeal microbiota in the presence of *B. pertussis* infection has been described in the mouse models only^15,16^. These studies evidenced a preventive action of the nasopharyngeal resident microbiota against *B. pertussis* colonization^15^, while the use of antibiotics produces the opposite effect, favoring *B. pertussis* colonization^16^.

Understanding the modulation of ecology of nasopharynx in humans with *B. pertussis* infection may help to understand determinants and potential diagnostic, preventive and therapeutic implications for this disease. In addition, no data are available on the changes that may derive from the simultaneous infection by *B. pertussis* and Rhinovirus.

In this study, we investigated the characteristics of nasopharyngeal microbiome in infants with *B. pertussis* and Rhinovirus simultaneous infection with those of either infection alone.

## Results

### Patients’ clinical features

Socio-demographic characteristics of patients included in the study are shown in Table 1.

**Table 1:** Characteristics of the patients (n = 54).

	Total (n = 54)	*B. pertussis* (n = 15)	Rhinovirus (n = 18)	Coinfection (n = 21)	*p* value
	n (%)	n (%)	n (%)	n (%)	
Males	31 (57.4)	9 (60.0)	10 (55.6)	12 (57.1)	0.967
Age (months)					
Median (IQR)	1.5 (1–3)	2.0 (1–3)	1.0 (1–2)	1.0 (1–3)	0.602
**Gestational age (weeks)**					
Median (IQR)	38 (37–40)	40 (37–41)	38 (37–38)	39 (38–40)	0.325
Gestational age*					
Preterm born (34–36 weeks)	10 (18.9)	3 (20.0)	4 (22.2)	3 (15.0)	0.844
Term born (37–42 weeks)	43 (81.1)	12 (80.0)	14 (77.8)	17 (85.0)	
**Weight (kg)**					
Median (IQR)	3.3 (3.0–3.5)	3.4 (3.1–3.7)	3.1 (3.0–3.5)	3.3 (3.0–3.5)	0.415
Type of delivery*					
Vaginal	27 (50.9)	6 (40.0)	9 (50.0)	12 (60.0)	0.501
Caesarean	26 (49.1)	9 (60.0)	9 (50.0)	8 (40.0)	
Type of feeding*					
Exclusive maternal	23 (43.4)	2 (14.3)	9 (50.0)	12 (57.1)	0.029
Mixed	13 (24.5)	3 (21.4)	4 (22.2)	6 (28.6)	
Formula	17 (32.1)	9 (64.3)	5 (27.8)	3 (14.3)	

*missing values for 1 patient.

The majority of patients were males (57.4%) with a median age of 1.5 months; patients with *B. pertussis* infection were slightly older than those with Rhinovirus infection or coinfection.

Children with coinfection were more likely breastfed compared to those with Rhinovirus even if the difference is small (57% vs 50% respectively, *p* = 0.029). On the other hand, children with *B. pertussis* infection were more likely formula-fed compared to those with coinfection (64% vs 14%, *p* = 0.029).

Table 2 shows the clinical features among the three groups. Cough, paroxysmal cough, whooping and apnea were more frequent in patients with *B. pertussis* infection. Cyanosis was occurred more frequently in the coinfection group by comparison to the Bp or Rv groups. In addition, the coinfection group more likely uses antibiotics before admission.

**Table 2:** Clinical features by group of infection (n = 54).

	Total (n = 54)		*B. pertussis *(n = 15)		Rhinovirus (n = 18)		Coinfection (n = 21)		*p* value
	n	%	n	%	n	%	n	%	
Cough	45	83.3	15	100.0	11	61.1	19	90.5	0.006
Cyanosis	28	51.9	9	60.0	5	27.8	14	66.7	0.040
Vomit	22	40.7	5	33.3	7	38.9	10	47.6	0.678
Paroxysmal cough	32	59.3	14	93.3	2	11.1	16	76.2	< 0.001
Apnea	30	55.6	11	73.3	5	27.8	14	66.7	0.014
Fever	12	22.2	1	6.7	6	33.3	5	23.8	0.181
Whooping	24	44.4	11	73.3	0	–	13	61.9	< 0.001
Antibiotics before admission	13	25.0	4	28.6	1	5.6	8	40.0	0.047
**Antibiotics at admission**									
No antibiotic	12	22.2	1	6.7	9	50.0	2	9.5	< 0.001
Macrolides	35	64.8	13	86.7	3	16.7	19	90.5	
Other	7	13.0	1	6.7	6	33.3	0	–	
Leukocytes plus lymphocytes*	18	35.3	5	33.3	3	17.7	10	52.6	0.089
Complications	7	13.0	3	20.0	1	5.6	3	14.3	0.457
C-Reactive Protein (CRP)	23	43.4	5	33.3	10	55.6	8	40.0	0.407

*White cell count > max for age and lymphocytes > 50%.

Dendrogram analysis, based on clinical features’ correlation, revealed that C-reactive protein (CRP), fever and antibiotic treatment at the admission (others’ than macrolides) were correlated together and constituted a separated cluster from the other clinical variables. The other features were subgrouped into: (i) apnea, cyanosis, macrolides’ assumption and antibiotics treatment before the admission, and (ii) delivery, feeding cough, paroxysmal cough and inspiratory stridor, complications, vomit and leukocytes plus lymphocytes count (Fig. S1).

### Nasopharyngeal microbiota profiling

A total of 2,393,694 sequencing reads were obtained from 54 nasopharyngeal aspirates, with a mean value of 44,328 sequences per sample. We identified an overall of 245 operational taxonomic units (OTUs), belonging to 15 phyla and 95 families.

To assess the overall differences of microbial community structures for the three groups, ecological parameters were based on alpha-diversity indexes (*i.e.*, Shannon, Chao1, and Observed species indexes).

**Figure 1:**
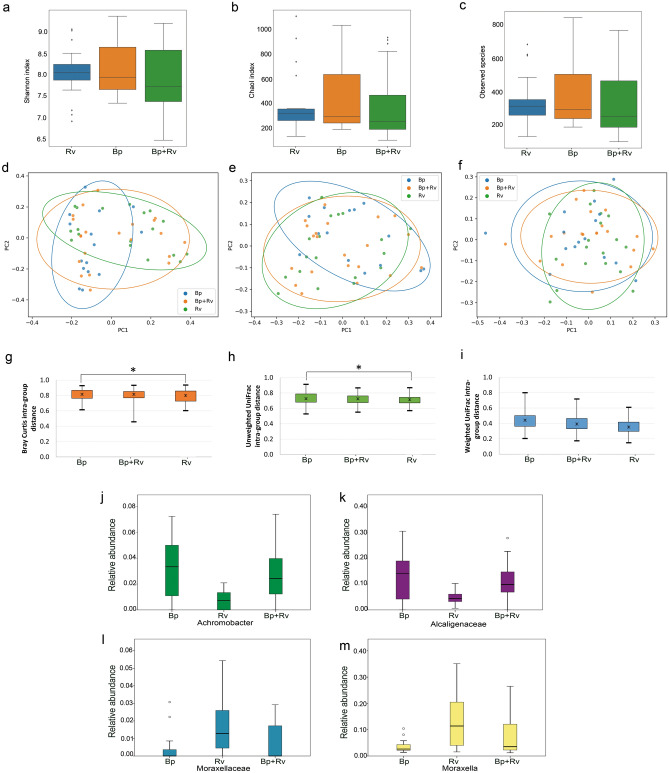
Microbiota diversity analysis. Alpha diversity analysis (**a**–**c**). Box plots show Shannon, Chao1, and Observed species indexes for each patients’ group. Microbiota beta diversity analysis (**d**–**f**). Principal coordinates analysis (PCoA) plot of bacterial beta-diversity based on Bray Curtis dissimilarity (**d**) and unweighted (**e**) and weighted (**f**) UniFrac phylogenetic distance. The plots show the first two principal coordinates (axes) of PCoA. Beta diversity distance box plots (**g**–**i**). Data are expressed as minimum, maximum and median values, and 25th and 75th percentiles values. The statistical significant differences are determined by permanova-pairwise tests and indicated by the star. Box plots of OTUs statistically significant by Kruskal–Wallis test (**j**–**m**). In y-axis are reported OTU’s relative abundances; in x-axis are reported median, minimum and maximum values, and the 25th and 75th percentile values.

Despite no statistical differences were resulted, samples belonging to Rv group showed higher values of all indexes followed by Bp samples and then Bp + Rv samples (Fig. 1, panels a–c).

Regarding beta diversity analysis, only Bray Curtis algorithm evidenced a statistically significant separation amongst groups (PERMANOVA *p* value ≤ 0.05), while phylogenetic unweighted and weighted UniFrac algorithms not (PERMANOVA *p* values > 0.05) (Fig. 1, panels d–f). Analyses of the intra-group distances revealed statistically significant differences between Bp and Rv for Bray–Curtis and Unweighted UniFrac metrics (Fig. 1, panels g–i).

The OTU distribution was investigated at the phylum and genus levels. At phylum level, Proteobacteria, Firmicutes, Actinobacteria and Bacteroidetes were the most representative phyla in the three groups (Fig. S2, Panel A). At genus level, Enterobacteriaceae, *Veillonella*, *Staphylococcus*, Gemellaceae, Alcaligenaceae and *Achromobacter* were increased in Bp ecosystem, while *Streptococcus* and *Moraxella* in Rv group (Fig. S2, Panel B).

Kruskal–Wallis test confirmed the higher abundance of *Achromobacter* and Alcaligenaceae in Bp group compared to the other groups (raw *p* value < 0.05) (Fig. 1, panels j and k), and the higher abundance of *Moraxella* and Moraxellaceae in Rv compared with others (raw *p* value < 0.05) (Fig. 1, panels l and m).

In the comparison amongst the three groups the principal component analysis (PCA) (Fig. S3, Panel a) and the partial least square (PLS) (Fig. S3, Panel b) analyses, performed on OTU’s profiles at genus levels, reveled a more separated Bp clusters compared to the other two groups. The same analyses, restricted to Bp and Rv comparison, revealed two separated PCA clusters (Fig. S3, Panel c) and none in PLS (Fig. S3, Panel d).

### Correlation between clinical features and nasopharyngeal microbiota global distribution

**Figure 2:**
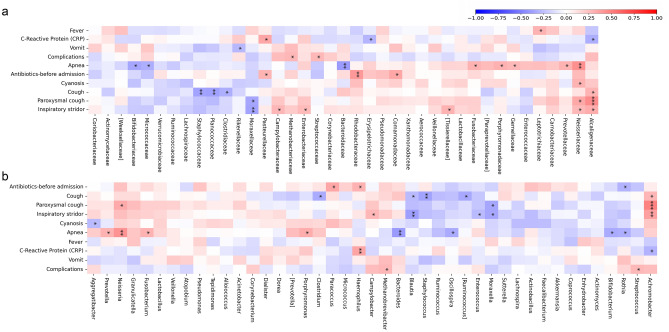
Pearson’s heat-map correlation. The Pearson’s correlation is calculated between clinical features and OTUs’ relative abundances at family (**a**) and genus (**b**) levels. The color scale represents the scaled level of each variable: red, positive correlation values; blue, negative correlation values. The stars indicate the statistically significant correlations (*p* value ≤ 0.05).

At family level, Pearson’s correlation revealed that all considered clinical features showed a correlation with a least one OTU. In particular, Alcaligenaceae was positively correlated with cough, paroxysmal cough and inspiratory stridor, while negatively with CRP. Moraxellaceae was negatively correlated with paroxysmal cough and inspiratory stridor (Fig. 2, panel a).

At genus level, antibiotic treatment before the admission was positively correlated with *Paracoccus* and *Haemophilus* and negatively with *Rothia*. Cough was negatively correlated with *Clostridium*, *Blautia*, *Staphylococcus* and *Ruminococcus* and positively with *Achromobacter*.

Paroxysmal cough was positively correlated with *Neisseria* and *Achromobacter* and negatively with *Moraxella*. Inspiratory stridor was positively correlated with *Campylobacter* and *Achromobacter* and negatively with *Blautia*, *Enterococcus* and *Moraxella*. Cyanosis was negatively correlated with *Aggregatibacter*. Apnea was positively correlated with *Prevotella*, *Neisseria*, *Fusobacterium* and *Porphyromonas* and negatively with *Bacteroides*, *Oscillospira*, *Bifidobacterium* and *Rothia*. CRP had positive correlation with *Haemophilus* and negative with *Achromobacter*. Finally, complications were positively related with *Methanobrevibacter* and *Streptococcus* (Fig. 2, panel b).

### Comparison between Bp- and Rv-related microbiota and patients’ clinical profiles

To focus onto microbiota specific features associated to *B. pertussis* or Rhinovirus infection and to highlight their correlations with patients' clinical traits, we focalized on Bp- and Rv-related microbiota, excluding the coinfection group (Bp + Rv).

**Figure 3:**
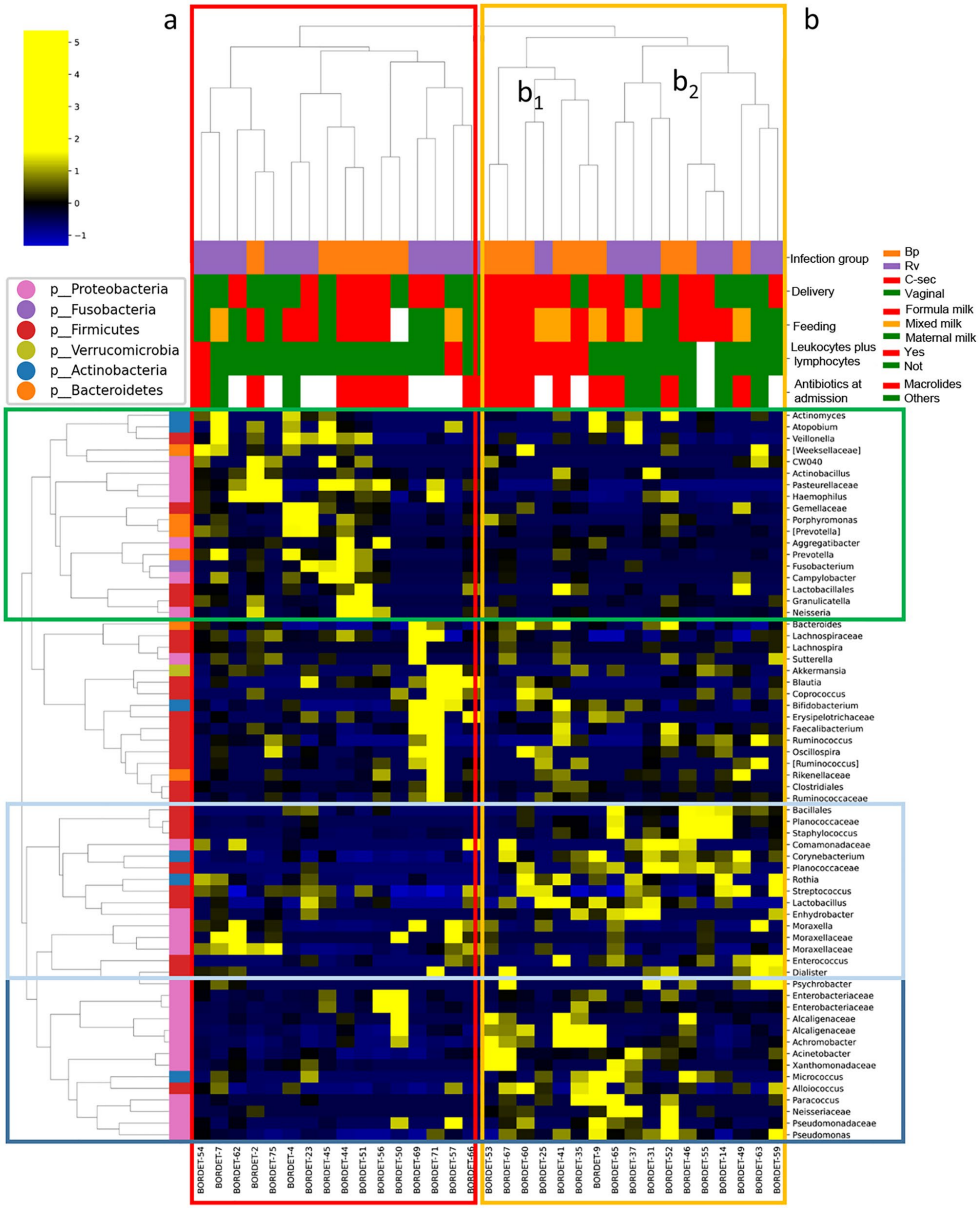
Hierarchical cluster of Bp and Rv patients according to OTU distribution at the genus level. The heat-map reports the hierarchical Ward-linkage clustering based on the Pearson’s correlation coefficient amongst OTUs at genus level. The color scale represents the scaled level of each variable: yellow, high level; blue, low level. The column bar is colored according to the subject category and clinical features’ groups. The left bar is colored according to the phylum level taxonomy.

The Pearson’s correlation performed on OTU profiles at genus level revealed two wide clusters. The first (a) was associated to Rv patients (9/16) with a nasopharyngeal microbiota profile rich in genera belonging to Actinobacteria, Bacteroidetes, Firmicutes and Proteobacteria, and related to low levels of leukocytes + lymphocytes (14/16) (Fig. 3). The second (b) was composed by two sub-clusters, namely b_1_ and b_2_. The cluster b_1_ was prevalently associated to Bp patients (6/7), characterized by high levels of leukocytes and lymphocytes, Caesarian section delivery and formula milk or mixed milk feeding and by a nasopharyngeal microbiota principally composed by Proteobacteria (Fig. 3). The patients in cluster b_2_, mostly Rv infected, was characterized by low level of leukocytes and lymphocytes, no antibiotic at the admission and not specific feeding and delivery modality and with a nasopharyngeal microbiota profile prevalently composed by Firmicutes and Proteobacteria (Fig. 3).

### Model classifications analysis

To investigate if nasopharyngeal microbiota profile could be predictive of a specific type of infection, we used the model classification analysis. This analysis, at genus level, revealed that the microbiota had capability to classify the 90% of the Bp- and Rv-related patients by the models Extra Trees Classifier, Gradient Boosting Classifier, Bagging Classifier and Decision Tree Classifier. A lower level of classification for Bp- (80%) was obtained compared to the classification of Rv-related patients (100%) (Table S1). Moreover, applying Random Forest Classifier and XGBRF Classifier, the microbiota profile reached the 100% of capability in correctly classifying both groups of patients.

**Figure 4:**
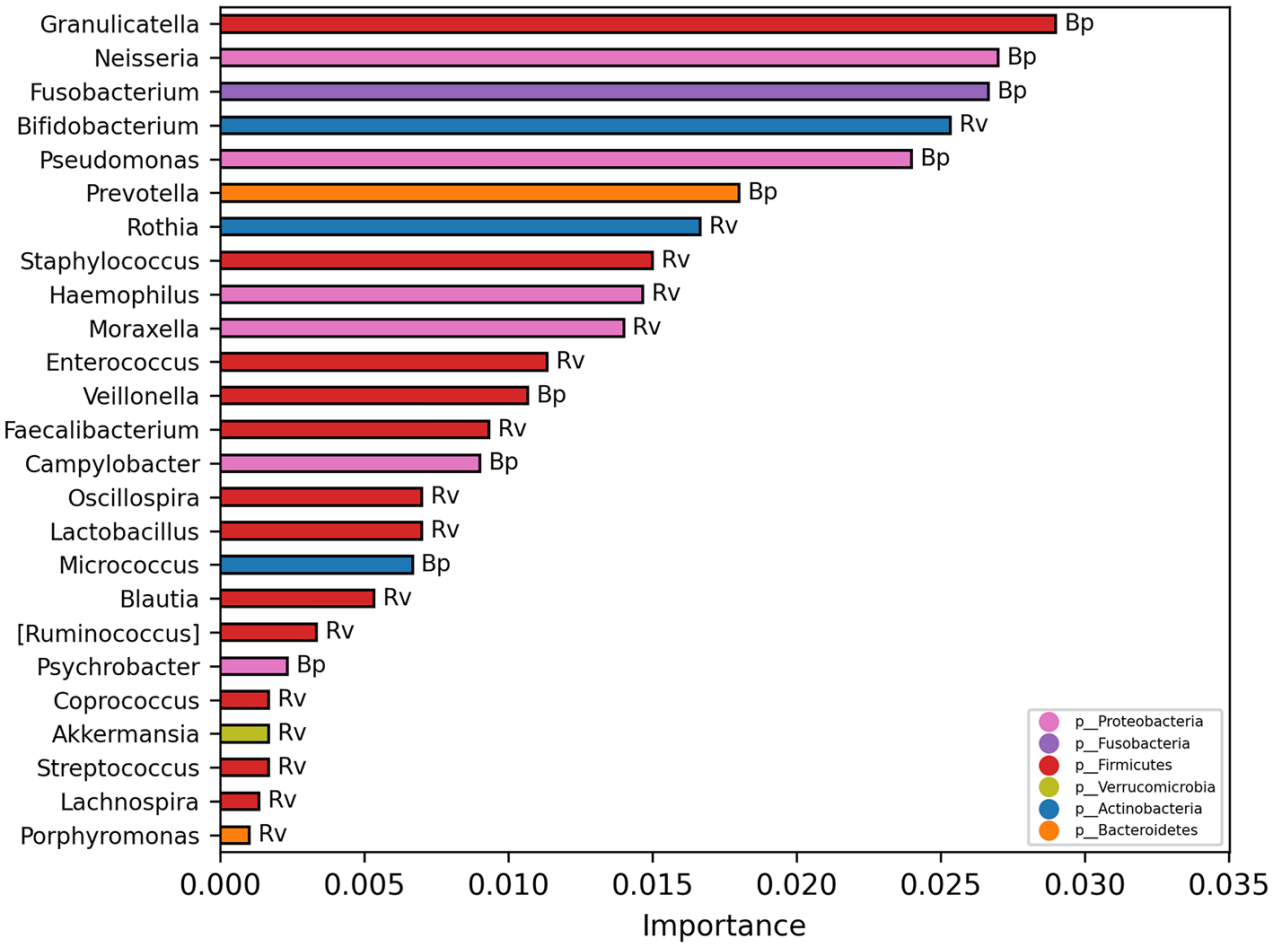
Important OTUs selected by model classification analysis. The bars represent the importance scores of each OTUs in the prediction of models.

In Fig. 4 were reported the OTUs selected by the model analysis and the respective contribution value (importance score) in the model prediction.

## Discussion

Our study describes for the first time the nasopharyngeal microbiota in children with pertussis.

We showed a different microbiota profiles consisting in the increase of Enterobacteriaceae, *Veillonella*, *Staphylococcus* Gemellaceae, Alcaligenaceae and *Achromobacter* in Bp group and of *Streptococcus* and *Moraxella* in Rv group.

The absence of comparison between *B. pertussis* infected patients with healthy subjects, due to the invasiveness of the nasopharyngeal aspirate in healthy infants, represents a limitation of this study. For this reason and in consideration of the high prevalence of cases of *B. pertussis*-Rhinovirus coinfection, we compared the nasopharyngeal microbiota profile of infants affected by single infection of *B. pertussis* or Rhinovirus with that of coinfected patients, who routinely underwent nasopharyngeal aspirate.

The nasopharyngeal microbiota of children is densely colonized by commensal bacteria, such as *Moraxella*, *Haemophilus*, *Streptococcus*, *Flavobacterium*, *Dolosigranulum*, *Corynebacterium*, *Neisseria* and *Fusobacterium*. However, *Streptococcus pneumoniae*, *Haemophilus influenzae* and *Moraxella catarrhalis* are considered potential airway pathogens^17^. When the nasopharyngeal eubiotic status is perturbed, these potentially pathogenic microorganisms can invade adjacent sites and cause different diseases^17–22^. It remains not clear which is the mechanism favoring pathogens’ colonization; however the interaction amongst multiple pathogens including viral coinfections, the progressive shift from health to disbiotic status and the effect of antibiotic administration could represent triggering factors^17,20,23^.

Based on our results, a reduction of nasopharyngeal microbial richness was evident in coinfection related microbiota compared to that related to single infections. The reduction of the diversity of the nasopharyngeal microbiota has been actually associated with the increase frequency of upper respiratory infections^4,24^. Moreover, considering the comparison between coinfected and Rv groups, this effect could also depend to macrolide intake. In fact, 90.5% of coinfected patients took macrolides respect to only 16.7% of Rv. On the contrary, also Bp patients took macrolides (87.6%), then the difference in microbiota diversity with coinfected patients could be driven by the different type of infection.

Analyzing the distance matrices and the composition of the microbiota, the co-infected microbiota profile was similar to both single infections' microbiota, even if with a higher similarity to the Rv group.

The comparison amongst the three groups revealed the increase of Alcaligenaceae and *Achromobacter* in the Bp infection group. *Achromobacter* spp., belonging to Alcaligenaceae family, are environmental microorganisms, causing a series of opportunistic illnesses, including respiratory diseases^25,26^. In our study, *Achromobacter* resulted related to cough, paroxysmal cough and inspiratory stridor, suggesting a role of this microorganism in the disease prognosis. Moreover, Moraxellaceae and *Moraxella* were increased in Rv infection group, as confirmed also by literature^27–29^. During Rv infection it has been described a colonization by as *S. pneumoniae*, *H. influenzae* and *M. catarrhalis*^3,13,30^. In particular, it seems that the nasopharyngeal colonization by *Streptococcus* and *Moraxella* precedes the Rv infection, probably due to the decreasing in ciliary function of epithelial cells and increasing of pro-inflammatory molecule release^30^. The simultaneous presence of Rv and *Moraxella* seems to be associated with severe lower respiratory infections, suggesting a correlation between specific viral/bacterial interactions and increase of disease severity^3^.

Evaluating the impact of delivery and feeding modality, count of leukocytes, administration of antibiotics it was possible to identify specific taxa as representative of Rv and Bp groups. Particularly, patients with vaginal delivery, maternal feeding, low count of leukocytes plus lymphocytes, low administration of antibiotics and rich microbiota profile resulted those infected by Rhinovirus. On the contrary, patients infected by *B. pertussis* were characterized by caesarian section delivery, formula milk feeding, high counts of leukocytes, antibiotic administration and high level of Proteobacteria. Thanks to these evidences we speculate that these conditions could shape the microbiota to be more prone to the airway infection by *B. pertussis.*

In conclusion, external factors since the first moments of life contribute to the alteration of nasopharyngeal microbiota, and this event could increase the susceptibility of the host to the pathogens' infections. In our opinion, when the infection is triggered, the presence of infectious agents could further modify the microbiota ecological niche favoring the overgrowth of other commensal bacteria that turn in pathobionts, hence contributing to the disease severity.

## Methods

### Study design and population

This study was performed at Bambino Gesù Children’s Hospital (OPBG), of Rome, Italy. From January 2016 to December 2019, all infants younger than 1 year of age, accessing the emergency room with symptoms compatible with pertussis, according to European Centre for Disease Prevention and Control (ECDC) case definition, were routinely screened for pertussis and respiratory viruses in the context of an enhanced surveillance program. A subset of patients with either *B. pertussis* (Bp) or Rhinovirus (Rv) or both infections (Bp + Rv), for whom a leftover of the nasopharyngeal aspirate was available for the analysis of the pharynx microbiota, was selected for this study. The study was conducted in accordance with the Declaration of Helsinki, and the protocol was approved by Bambino Gesù Children’s Hospital Ethics Committee (1355_OPBG2017). Informed consent was obtained from the parents or legal guardians of all participants.

### Data collection and laboratory confirmation of pertussis

Sociodemographic and clinical data were collected for each patient. Nasopharyngeal aspirate were collected within 24 h of hospital admission and processed immediately, or stored at − 80 °C until analysis. Two-hundred μl of nasopharyngeal sample were used for nucleic acids extraction by the EZ1 Virus Mini Kit v. 2.0 on the EZ1 Advanced XL platform (Qiagen, GmbH, Hilden, Germany).

Bordetella Real Time (RT) PCR kits were used to investigate the presence of *B. pertussis*, amplifying the inter-space (IS) 481 target (Bordetella R-gene™ assay; Argene, Biomerieux, Marcy l’Etoile, France). To prevent misdiagnosis of *Bordetella holmesii* as *B. pertussis*, all samples positive for *B. pertussis* were confirmed by the amplification of *ptxP* (promoter of pertussis toxin) locus by a RT-PCR assay specific for *B. pertussis*. Moreover, culture for *B. pertussis* was also performed, using Regan-Lowe and Bordet-Gengou selective culture media at 37 °C under aerobic conditions. Nasopharyngeal aspirates were also processed by a multiplex RT-PCR using a specific panel detecting the 16 following viruses: Respiratory syncytial virus (RSV) A and B; Influenza virus A and B; Coronaviruses OC43, 229E, cNL63, and HUK1; adenovirus; human Rhinovirus (hRV); parainfluenza virus 1–2–3–4; human Metapneumovirus-hMPV and human Bocavirus-hBoV.

### 16S rRNA targeted-metagenomics of nasopharyngeal microbiota

DNA from 200 µl of nasopharyngeal samples was automatically extracted using the EZ1 DNA Tissue Kit and biorobot EZ1 extractor following manufacturer’s instructions (Qiagen, Hilden, Germany). The V3-V4 region of 16S rRNA gene (~ 460 bp) was amplified by PCR using the primers 16S_F 5′-(TCG TCG GCA GCG TCA GAT GTG TAT AAG AGA CAG CCT ACG GGN GGC WGC AG)-3′ and 16S_R 5′-(GTC TCG TGG GCT CGG AGA TGT GTA TAA GAG ACA GGA CTA CHV GGG TAT CTA ATC C)-3′ as reported in the MiSeq rRNA Amplicon Sequencing protocol (Illumina, San Diego, CA). The first amplification was carried out setting up the following conditions: initial denaturation at 95 °C for 3 min, 32 cycles of denaturation at 95 °C for 30 s, annealing at 55 °C for 30 s, and extension at 72 °C for 30 s, and a final extension step at 72 °C for 5 min, with Fast Start Hifi Taq kit (Roche Diagnostics, Mannheim, Germany). DNA amplicons were cleaned with 20 ul of KAPA Pure Beads (Roche Diagnostics, Mannheim, Germany) and tagged with unique index combinations using Nextera technology in a second amplification step. Following steps consisted of cleaning of the final library, quantification with Quant-iT™ PicoGreen® dsDNA Assay Kit (Thermo Fisher Scientific, Waltham, MA) and dilution to 4 nM concentrations. Pooling, denaturation and dilution to 7 pM were performed before the sequencing on an Illumina MiSeqTM platform (Illumina, San Diego, CA, United States) where paired-end reads of 300 base-length were generated.

All raw sequences have been archived in NCBI database: PRJNA730698 (https://www.ncbi.nlm.nih.gov/bioproject).

### Biocomputational and statistical analyses

Illumina Miseq reads were checked for quality, length and chimera presence using the Qiime v1.9 pipeline^31^, and USEARCH algorithms^32^. Then, sequences were organized into Operational Taxonomic Units (OTUs) with a 97% of clustering threshold of pairwise identity by UCLUST with open reference method^32^. PyNAST v.0.1 software^31^ was used to carry out a multiple sequence alignment against Greengenes 13_08 database with a 97% similarity for bacterial sequences and to build a phylogenetic tree^33^. Alpha diversity was performed by scikit-bio (http://scikit-bio.org/) of Python 3 package using Shannon index, Chao1 estimator, and observed species indices, and the *p* value for group comparisons was determined by analysis of variance (ANOVA). Beta diversity analysis of samples, based on phylogenetically informed weighted and unweighted Unifrac distance matrices^34^ and Bray Curtis matrices, was used and graphed by principal coordinate analysis (PCoA) plots. The association between the covariates and beta diversity measures was assessed by permutational analysis of variance (PERMANOVA)^35^.

Categorical variables were tabulated as frequencies and percentages and compared using Pearson’s Chi square test.

OTUs were filtered to select only the features of interest by retaining OTUs with interquartile range (IQR) ≠ 0 and OTUs present in at least 25% of the samples.

The non-parametric Mann–Whitney U-test, Wilcoxon signed-rank, Kruskal–Wallis test, corrected for FDR *p* value ≤ 0.05, were used to compare OTUs relative abundance amongst groups.

Both the OTUs and clinical variables (considered both raw values and categorical variables) were clustered using the Pearson’s correlation coefficient as the distance measure. In order to correlate clinical variables and OTUs, the Pearson’s coefficient with relative *p* value was calculated and represented by heat map.

Multiple machine learning (ML) was trained for the classification tasks. The pipeline consisted of a tenfold cross-validation with a train-test split of 70–30%. To evaluate the model, the global and the single class accuracies were considered. The tested models were: Logistic Regression, SGD Classifier, Logistic Regression CV, Hist Gradient Boosting Classifier, Random Forest Classifier, Extra Trees Classifier, Gradient Boosting Classifier, Bagging Classifier, Ada Boost Classifier, XGB Classifier, XGBRF Classifier, MLP Classifier, Linear SVC, SVC, Gaussian NB, Decision Tree Classifier, Quadratic Discriminant Analysis, K Neighbors Classifier, Gaussian Process Classifier.

## Supplementary Information

Supplementary Information.
