# Supplementary material for: Nasopharyngeal microbiota in hospitalized children with Bordetella pertussis and Rhinovirus infection
Source: Sci Rep. 2021 Nov 24;11:22858. doi: 10.1038/s41598-021-02322-y (PMC8613181; doi:10.1038/s41598-021-02322-y)
Supplement: Supplementary file 1 — Supplementary Information. [file 41598_2021_2322_MOESM1_ESM.docx]

**Supplementary Materials**

**
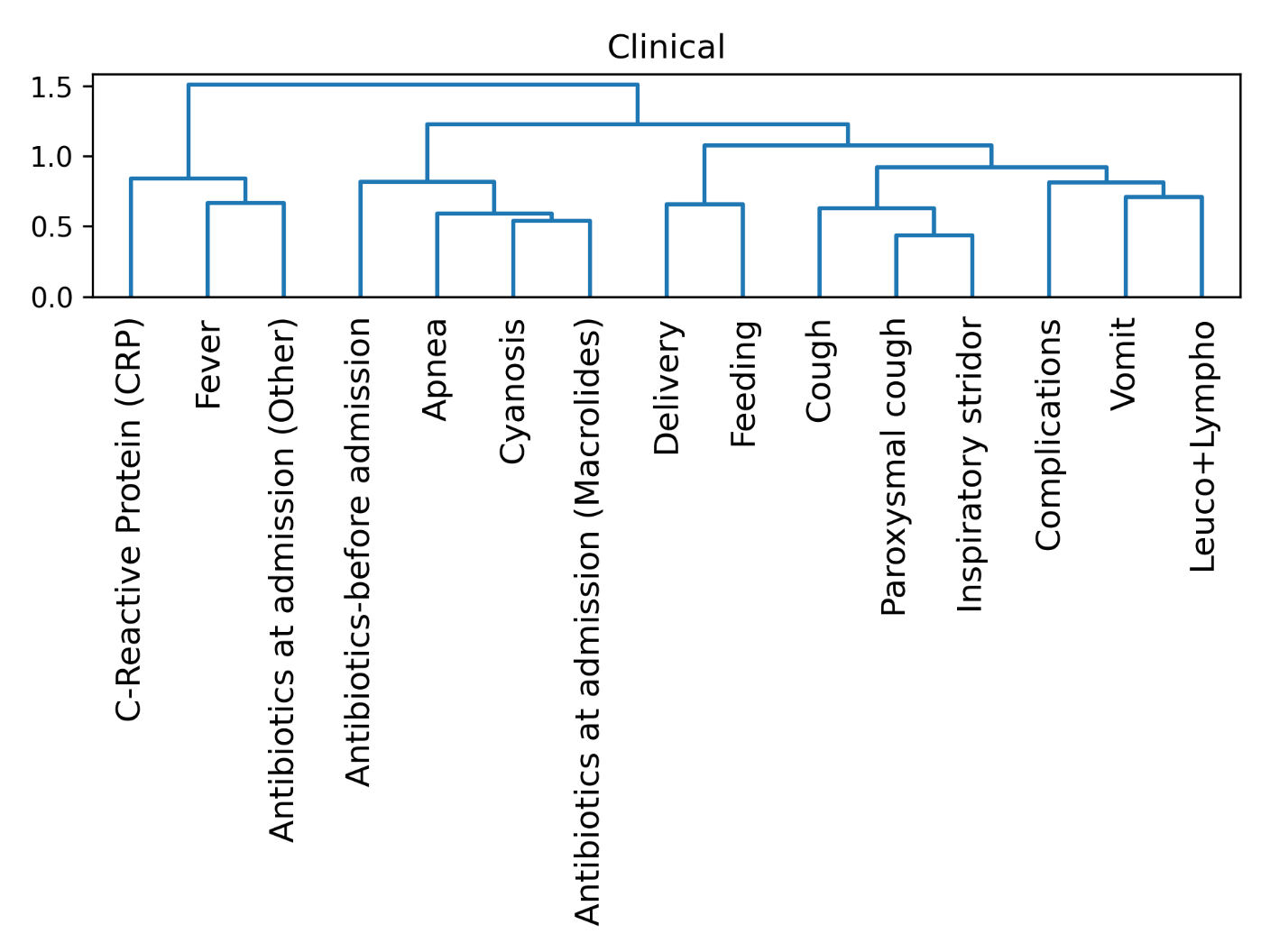
**

**Figure S1. Dendrogram of clinical patients’ profiles.** The clinical features are at the bottom of the dendrogram. The clinical features' clusters are formed by joining similar clinical patients’ profiles. The vertical axis refers to a distance measure calculated on patients' clinical profile similarity/dissimilarity.


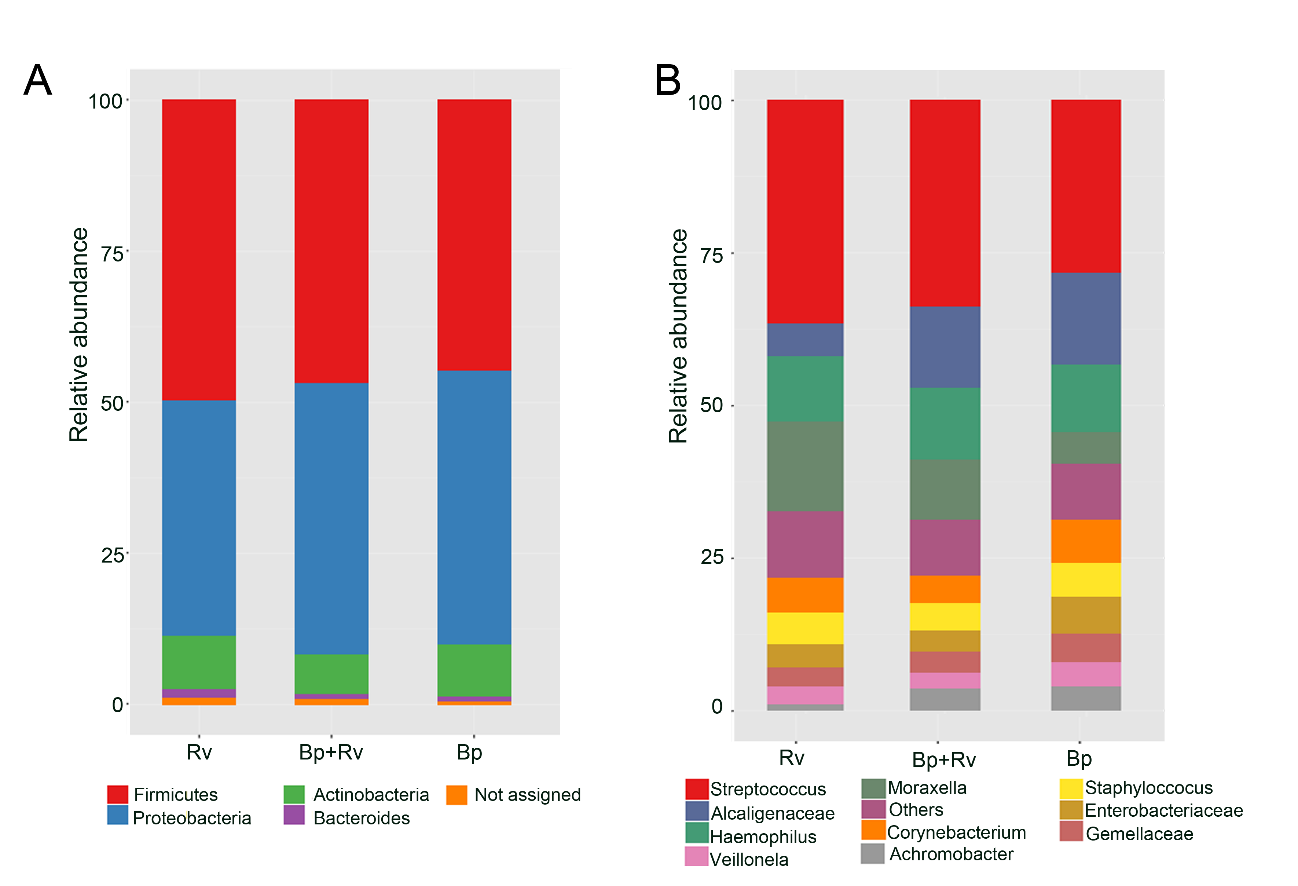


**Figure S2. Microbiota composition. (A-B) Histograms of operational taxonomic units (OTUs) grouped in phyla (A) and in families/genera (B) of the Rhinovirus (Rv), *B. pertussis* (Bp), and coinfection (Bp+Rv) groups.** Each color in the bars represents the percentage of relative abundance for each taxa.


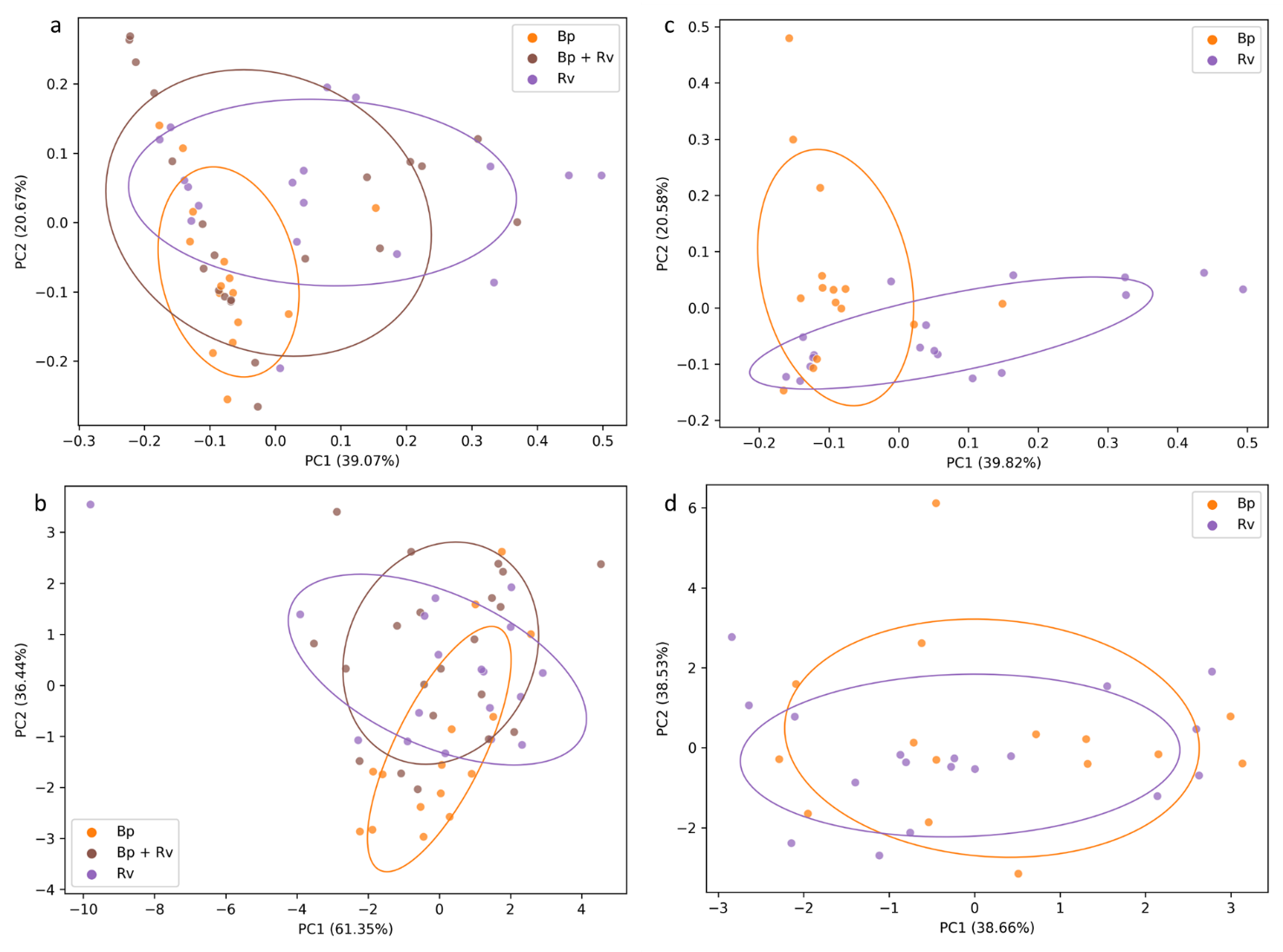


**Figure S3. Principal component analysis (PCA) (panels a and c) and partial least square (PLS) (panels b and d) score plots.** The first two principal components (PCs) are plotted and colored according to Bp, RV and Bp+RV and to Bp and RV, respectively. PCA and PLS analyses were performed on OTU’s profiles at genus levels. The variance percentage of each component is reported in each axis.

**Table S1.** Classification models applied to discriminate Bp and Rv patients on nasopharyngeal microbiota composition at genus level. In bold are reported the models with the higher classification scores.

| **Model** | **Score** | **Score Bp** |  | **Score Rv** |
| --- | --- | --- | --- | --- |
| Dummy Classifier | 0.5 | 0 |  | 1 |
| Logistic Regression | 0.7 | 0.6 |  | 0.8 |
| SGD Classifier | 0.7 | 0.8 |  | 0.6 |
| Logistic Regression CV | 0.7 | 0.6 |  | 0.8 |
| Hist Gradient Boosting Classifier | 0.5 | 0 |  | 1 |
| **Random Forest Classifier** | **1** | **1** |  | **1** |
| **Extra Trees Classifier** | **0.9** | **0.8** |  | **1** |
| **Gradient Boosting Classifier** | **0.9** | **0.8** |  | **1** |
| **Bagging Classifier** | **0.9** | **0.8** |  | **1** |
| Ada Boost Classifier | 0.5 | 0.6 |  | 0.4 |
| **XGB Classifier** | **0.8** | **0.8** |  | **0.8** |
| **XGBRF Classifier** | **1** | **1** |  | **1** |
| MLP Classifier | 0.6 | 0.6 |  | 0.6 |
| Linear SVC | 0.8 | 1 |  | 0.6 |
| SVC | 0.7 | 0.8 |  | 0.6 |
| Gaussian NB | 0.8 | 1 |  | 0.6 |
| **Decision Tree Classifier** | **0.9** | **0.8** |  | **1** |
| Quadratic Discriminant Analysis | 0.6 | 0.2 |  | 1 |
| K Neighbors Classifier | 0.7 | 0.8 |  | 0.6 |
| Gaussian Process Classifier | 0.7 | 0.6 |  | 0.8 |
